# Two different genetic etiologies for tuberous sclerosis complex (TSC) in a single family

**DOI:** 10.1002/mgg3.1296

**Published:** 2020-05-08

**Authors:** Kate Mowrey, Mary Kay Koenig, Charles A. Szabo, Joshua Samuels, Shannon Mulligan, Deborah A. Pearson, Hope Northrup

**Affiliations:** ^1^ Department of Pediatrics Division of Medical Genetics McGovern Medical School University of Texas Health Science Center at Houston Houston TX USA; ^2^ Department of Pediatrics Division of Child and Adolescent Neurology McGovern Medical School University of Texas Health Science Center at Houston Houston TX USA; ^3^ Department of Neurology and South Texas Comprehensive Epilepsy Center University of Texas Health Science Center at San Antonio TX USA; ^4^ Department of Pediatrics Division of Nephrology and Hypertension McGovern Medical School University of Texas Health Science Center at Houston Houston TX USA; ^5^ Department of Obstetrics, Gynecology and Reproductive Sciences Division of Genetic Counseling McGovern Medical School University of Texas Health Science Center at Houston Houston TX USA; ^6^ Department of Psychiatry and Behavioral Sciences McGovern Medical School at University of Texas Health Science Center at Houston Houston TX USA

**Keywords:** genetic counseling, genetic testing, *TSC2*, tuberous sclerosis complex

## Abstract

**Background:**

Tuberous sclerosis complex (TSC) is an autosomal dominant genetic condition that involves abnormalities of the skin, hamartomas in the heart, brain, and kidneys, seizures, as well as TSC‐associated neuropsychiatric disorders (TAND). About 90%–95% of individuals with TSC will have an identifiable pathogenic variant in either *TSC1* or *TSC2*. We present here two family members with clinical diagnoses of TSC that were later determined to be due to two different genetic etiologies.

**Methods:**

A 2‐year‐old Caucasian female (Patient 1) was born to non‐consanguineous healthy parents and was determined to have a clinical diagnosis of TSC at 2 months old. Her paternal great‐uncle (Patient 2) was also known to have a clinical diagnosis of TSC. Sequencing and deletion/duplication analysis for *TSC1* and *TSC2* were performed on both individuals.

**Results:**

Mutation analysis revealed that both Patient 1 and Patient 2 had identifiable pathogenic variants in *TSC2*. Patient 1 had c.4800_4801delTG (p.Cys1600Trpfs*2), while Patient 2 had c.4470_4471delinsTT (p.Glu1490_Lys1491delinsAsp*).

**Conclusion:**

To our knowledge, our clinical report is of significance as it is the third kindred to be identified with affected members with two distinct genetic etiologies for TSC. Our case report highlights the importance of incorporating genetic testing into the clinical evaluation for individuals with features suggestive of TSC.

## Graphical abstract

Our clinical report is of significance as no other family has been reported in the medical literature with two clinical individuals with tuberous sclerosis complex (TSC) caused by two distinct genetic etiologies. Patient 2 is the paternal great‐uncle to Patient 1, yet they have different pathogenic variants in *TSC2*. This report highlights the importance of incorporating genetic testing into the clinical evaluation for individuals with features suggestive of TSC.

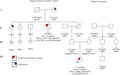

## 1 INTRODUCTION

Tuberous sclerosis complex (TSC) is an autosomal dominant genetic disorder that involves abnormalities of the skin, hamartomas in the heart, brain, and kidneys, seizures, as well as TSC‐associated neuropsychiatric disorders (TAND). The estimated incidence is 1 per 6,000 to 10,000 live births (Northrup et al., 2013). TSC has significant inter‐ and intrafamilial variable expressivity of the associated clinical characteristics, leading to a wide spectrum of disease severity (Au et al., 2007; Northrup, Koenig, Pearson, & Au, 2018; Sancak et al., 2005). The genetic etiology of TSC stems from heterozygous pathogenic variants in *TSC1* (MIM: 605284) or *TSC2* (MIM: 191092) that encode for the proteins, hamartin and tuberin, respectively (European Chromosome 16 Tuberous Sclerosis Consortium, 1993; van Slegtenhorst et al., 1997). Both *TSC1* and *TSC2* are tumor suppressors that aid in the regulation of cell growth and proliferation within the mechanistic target of rapamycin (mTOR) complex 1 (Castro, Rebhun, Clark, & Quilliam, 2003; Harris & Lawrence, 2003; Li, Corradetti, Inoki, & Guan, 2004; Van Slegtenhorst et al., 1998). Therefore, the loss of function in either gene leads to a reduction of functional protein, yet enough protein remains to regulate cell growth and proliferation. The loss of the remaining copy of *TSC1* or *TSC2* from a second somatic pathogenic variant consequently results in inadequate production of hamartin or tuberin. The lack of functional protein leads to uncontrolled cell growth and proliferation that contributes to the manifestation of numerous hamartomas (Au, Williams, Gambello, & Northrup, 2004; Northrup et al., 2018). Additionally, *TSC2* pathogenic variants are more likely to result in a more severe clinical picture and represent the majority of de novo simplex cases in comparison to pathogenic variants in *TSC1* (Au et al., 2007). Currently, an individual can receive a diagnosis of TSC based on meeting clinical criteria and/or molecular genetic testing that identifies a pathogenic variant in either *TSC1* or *TSC2* (Northrup et al., 2013). Often times, the benefit of genetic testing is the opportunity to perform family studies in order to differentiate between de novo or inherited cases.

There are two previous reports of kindreds with more than one pathogenic variant causative of TSC (Le Caignec et al., 2009; Osborne et al., 2000; Webb & Osborne, 1991). Le Caignec et al., 2009 reported a family with three independent pathogenic variants in *TSC2*. Furthermore, Webb and Osborne (1991) reported a family suggestive of non‐penetrance in TSC with an affected grandfather and affected grandson. Osborne et al., 2000 revisited this family and performed genetic testing that revealed two different genetic etiologies within the family. The grandson had a pathogenic variant in *TSC2* while other affected relatives had pathogenic variants in *TSC1*.

Here, we report a family with two members who have clinical diagnoses of TSC that were later determined to be caused by different genetic etiologies. Patient 2 is the great paternal uncle to Patient 1. Our clinical report is of significance as it is the third kindred to be identified with affected members with two distinct genetic etiologies for TSC.

## 2 CLINICAL REPORT

**FIGURE 1:**
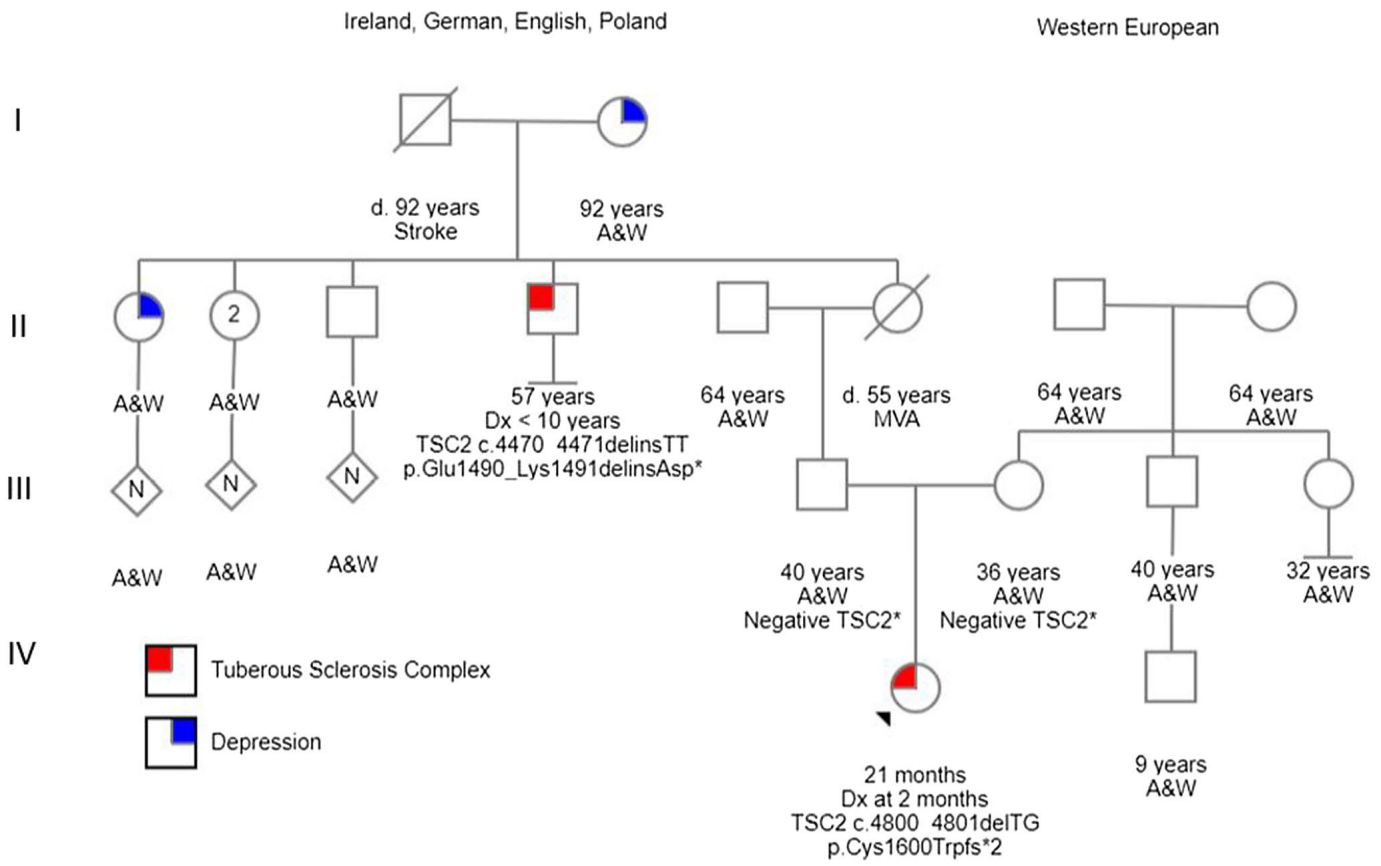
Pedigree for Patient 1 and Patient 2 demonstrates the relatedness as well as lists their two distinct genetic etiologies. Reference transcript for *TSC2* is NM_000548.3. A&W, Alive and Well; MVA, Motor Vehicle Accident

Patient 1 (IV‐1, Figure 1) was conceived through in vitro fertilization (IVF) and had prenatal genetic screening (PGS) performed prior to implantation. Prior to starting IVF, parents had carrier screening performed that was negative and required no follow‐up. Overall, the pregnancy did not have any complications or exposures. The 20‐week anatomy scan did not identify any abnormalities. Secondary to being an IVF pregnancy, a fetal echocardiogram was performed at 22 weeks and did not identify any structural or anatomical abnormalities in the heart. Patient 1 was born at 39w6d as a spontaneous vaginal delivery. Her APGARS were 2, 5, 6, and 7 at 1, 5, 10, and 15 min, respectively. At birth, Patient 1 was in respiratory distress and was given positive pressure ventilation (PPV), transitioned to continuous positive airway pressure (CPAP), and was briefly brought to the neonatal intensive care unit (NICU). Her birth weight was 3,251 g (37th percentile), birth length was 52.5 cm (82nd percentile), and head circumference at birth was 34 cm (29th percentile). She was weaned off CPAP and was discharged on day of life 3. At 2 months old, her pediatrician noted that her left arm was swollen and was concerned for an underlying cardiovascular issue. At that time, she was referred to cardiology for an evaluation. A subsequent echocardiogram revealed five non‐obstructing cardiac rhabdomyomas. Given this finding, Patient 1 was referred to The University of Texas Health Science Center at Houston's TSC Center of Excellence for further evaluation.

At 2 months old, Patient 1 was evaluated by genetics and given a clinical diagnosis of TSC. The two major clinical features that led to her diagnosis were multiple cardiac rhabdomyomas and three hypomelanotic macules that were greater than 5 mm in diameter. After the initial consultation, an order was placed for *TSC1/TSC2* sequencing and deletion/duplication analysis, a brain MRI, and an abdominal MRI, along with referrals to dermatology and ophthalmology. At 4 months, ophthalmology's evaluation identified bilateral retinal astrocytic hamartomas. The astrocytomas have remained stable and did not affect vision. Additionally, Patient 1 has hyperopia and astigmatism. At 6 months, dermatology confirmed the presence of hypomelanotic macules as well as a hemangioma located on her left chest and lymphedema of the left arm. The abdominal MRI revealed punctate bilateral renal cysts and no angiomyolipomas. The brain MRI identified numerous cortical tubers and multiple subependymal nodules with no hydrocephalus as well as no suggestion of a subependymal giant cell astrocytoma (SEGA). Genetic testing identified a pathogenic variant in *TSC2*, c.4800_4801delTG (p.Cys1600Trpfs*2). The reference transcript used for *TSC2* was NM_000548.3. Parental testing was performed indicating that the variant was de novo. The commercial laboratory performing the parental testing provides full gene sequencing and deletion/duplication analysis for all samples undergoing familial variant analysis. Therefore, it is known that the parents of Patient 1 do not have any additional variants within *TSC2*.

Patient 1 and her family elected to participate in a clinical trial that involved serial electroencephalograms (EEGs) and neurodevelopmental screening. She had serial EEGs every 6 weeks starting at 3 months of age. Patient 1’s EEGs did not show any epileptiform activity. At 12 months, Patient 1 presented to the hospital with a febrile upper respiratory infection and went into status epilepticus twice. She was subsequently started on vigabatrin. At her 6‐ and 12‐month clinical evaluations, she was obtaining her developmental milestones appropriately for her age. At Patient 1’s neurodevelopmental screening at 24 months, the Bayley‐III was administered by our neuropsychology team. The Bayley‐III is a widely used measure of infant and toddler development with high reliability and validity that assesses functional status in several domains, including language, motor, and cognition. Her composite scores for the Bayley‐III language, motor, and cognitive were 86, 85, and 100, respectively. At this time, her language and motor are in the low average range while her cognition is in the average range relative to other toddlers her age.

Patient 2 (II‐4, Figure 1) is a 57‐year‐old male who was born after an uncomplicated, full‐term pregnancy and vaginal delivery. He received a clinical diagnosis of TSC prior to 10 years of age. At 2 years of age, Patient 2 was misdiagnosed with a cataract in his left eye. It was later determined to be a unilateral retinal hamartoma. He continues to follow with ophthalmology and by report the retinal hamartoma has remained stable over his lifetime. The dermatological features consistent with his diagnosis of TSC include facial angiofibromas and multiple hypomelanotic macules that have been present since childhood.

Patient 2 experienced his first seizure prior to three months of age and was given a diagnosis of epilepsy before starting kindergarten. Subsequently, Patient 2 was started on antiepileptic drugs (AEDs). Per report, his seizures were well‐controlled on AEDs during childhood. Imaging studies performed in childhood identified six subependymal nodules (SENs) that have remained stable throughout his life. Patient 2 regularly took carbamazepine and phenobarbital from 14 to 18 years of age. He then developed daily headaches, which was due to a SEGA that obstructed cerebrospinal fluid flow through the foramen of Monro. Over three decades ago, at the age of 19 years, Patient 2 underwent a right frontal craniotomy for a tumor resection. Additionally, a ventriculoperitoneal shunt was placed with resolution of increased intracranial pressure and hydrocephalus. In late 2015, Patient 2 was converted to lamotrigine monotherapy due to the potential adverse effects of prolonged use of carbamazepine. His last seizure was in August 2016. Patient 2’s brain MRI from 2019 demonstrated right frontal encephalomalacia related his previous surgery, enhancing and calcified SENs bilaterally as well as bilateral multiregional hamartomas. Patient 2 was noted to have hypertension around 33 years of age and was subsequently given antihypertensive medication. Additionally, he was hospitalized for heart palpitations in his early 30s. During the evaluation, a concern was raised regarding the amount of fluid his body was retaining. Subsequent abdominal imaging studies were performed identifying angiomyolipomas. Genetic testing performed on Patient 2 in January 2019 revealed a pathogenic variant in *TSC2,* c.4470_4471delinsTT (p.Glu1490_Lys1491delinsAsp*). The reference transcript used for *TSC2* was NM_000548.3. Parental testing was not performed because Patient 2’s father is deceased and his mother was not interested in genetic testing at the time; therefore, we are uncertain of de novo versus inherited status.

Patient 2’s gross motor and speech development was reported as normal. Academically, Patient 2 took mainstream coursework throughout school with an Individualized Educational Plan (IEP). During the school year, he received speech therapy every week from first grade through sixth grade. He graduated high school and did not pursue any higher level education. Patient 2 struggled with anxiety since childhood, but reports an increase in severity in his mid‐40s secondary to a death in the family. Around the age of 50 years, he started on citalopram. Otherwise, there are no additional mental health concerns.

## 3 DISCUSSION

Our case report highlights the importance of incorporating genetic testing into the clinical evaluation for individuals with features suggestive of TSC. In the case of Patient 1 and Patient 2, genetic testing was able to determine that these two cases of TSC, regardless of their familial relationship, were due to different pathogenic variants both within *TSC2*. It is important to note that at the time of Patient 2’s diagnosis, genetic testing would not have been available. Additionally, follow‐up parental studies for Patient 1 allowed us to genetically confirm the presence of a de novo pathogenic variant in *TSC2*. This was of great importance to this family as they are planning future rounds of IVF. Knowing the de novo nature of Patient 1’s pathogenic variant provided the parents with a known recurrence risk of <1% in all future pregnancies. In general, parental testing allows for the identification of other affected family members who had previously been missed clinically. Without parental testing, appropriate recurrence risks cannot be communicated to the parents and affected individuals could delay the initiation of proper surveillance. Furthermore, in this case, genetic testing eliminated any concern from the clinician's side of variable expressivity being a possible explanation for the seemingly negative paternal family history.

As genetic testing has evolved, the International Tuberous Sclerosis Complex Consensus Group has appropriately reflected it in their published guidelines. In 2012, the Tuberous Sclerosis Complex Surveillance and Management recommendations were updated to include the identification of a heterozygous pathogenic variant in either *TSC1* or *TSC2* by molecular genetic testing as meeting criteria for a definitive diagnosis of TSC. Notably, the change from the Sanger sequencing to next‐generation sequencing (NGS) platform has led to the discovery of variants that had previously been undetectable. In comparison to Sanger sequencing, NGS has higher sequencing depths, higher power to identify novel variants, higher mutation resolution, and higher throughput with sample multiplexing. As a result, we are now able to detect genetic changes, such as low‐level mosaicism and deep intronic variants that would not have been identified using Sanger sequencing. With the availability of the NGS platform, some patients previously considered to be no mutation identified (NMI) on the Sanger sequencing platform have been able to uncover the genetic etiology of their TSC after receiving updated genetic testing (Tyburczy et al., 2015). Given the recent ability to detect deep intronic variants on the NGS platform, there should be standardization among all commercial laboratories in their identification and classification of deep intronic variants to further improve the utility of these variants in the clinical setting.

As genetic testing continues to improve with time, we strive to reduce the number of individuals within the NMI category, enhance genotype–phenotype correlations, and establish protocols to properly evaluate for mosaicism. The case of Patient 1 and Patient 2 reinforces the utility of genetic testing in the clinical setting and the impact it can have on multiple generations of a family. These future goals of genetic testing within the realm of TSC are targeted to improve the quality of care and provide better prognostic information.

## CONFLICT OF INTEREST

The authors declare no financial or otherwise relevant conflict of interest related to this manuscript.

## Data Availability

The data that support the findings of this study are available on request from the corresponding author. The data are not publicly available due to privacy or ethical restrictions.
